# Evaluation of Efficacy of Surface Coated versus Encapsulated Influenza Antigens in Mannose–Chitosan Nanoparticle-Based Intranasal Vaccine in Swine

**DOI:** 10.3390/vaccines12060647

**Published:** 2024-06-11

**Authors:** Dina Bugybayeva, Ekachai Dumkliang, Veerupaxagouda Patil, Ganesh Yadagiri, Raksha Suresh, Mithilesh Singh, Jennifer Schrock, Sara Dolatyabi, Olaitan C. Shekoni, Hadi M. Yassine, Praneet Opanasopit, Harm HogenEsch, Gourapura J. Renukaradhya

**Affiliations:** 1 Center for Food Animal Health, Department of Animal Sciences, The Ohio State University, Wooster, OH 44691, USA; bugybayeva.1@buckeyemail.osu.edu (D.B.); ekachai.d@psu.ac.th (E.D.); vkpatil13@gmail.com (V.P.); yadaigiri.1@osu.edu (G.Y.); suresh.138@buckeyemail.osu.edu (R.S.); drmithileshsingh@yahoo.com (M.S.); schrock.57@osu.edu (J.S.); dolatyabi.1@buckeyemail.osu.edu (S.D.); shekoni.2@buckeyemail.osu.edu (O.C.S.); 2 Drug Delivery System Excellence Center (DDSEC), Department of Pharmaceutical Technology, Faculty of Pharmaceutical Sciences, Prince of Songkhla University, Songkhla 90110, Thailand; 3 Pharmaceutical Development of Green Innovations Group (PDGIG), Faculty of Pharmacy, Silpakorn University, Nakhon Pathom 73000, Thailand; opanasopit_p@su.ac.th; 4 Biomedical Research Center, Qatar University, Doha 2713, Qatar; hyassine@qu.edu.qa; 5 Department of Comparative Pathobiology, College of Veterinary Medicine, Purdue University, West Lafayette, IN 47907, USA; hogenesc@purdue.edu

**Keywords:** intranasal vaccination, nanoparticles, H1N2 swine influenza virus, adjuvanted vaccine, mannose–chitosan, cellular immunity

## Abstract

This study focuses on the development and characterization of an intranasal vaccine platform using adjuvanted nanoparticulate delivery of swine influenza A virus (SwIAV). The vaccine employed whole inactivated H1N2 SwIAV as an antigen and STING-agonist ADU-S100 as an adjuvant, with both surface adsorbed or encapsulated in mannose–chitosan nanoparticles (mChit-NPs). Optimization of mChit-NPs included evaluating size, zeta potential, and cytotoxicity, with a 1:9 mass ratio of antigen to NP demonstrating high loading efficacy and non-cytotoxic properties suitable for intranasal vaccination. In a heterologous H1N1 pig challenge trial, the mChit-NP intranasal vaccine induced cross-reactive sIgA antibodies in the respiratory tract, surpassing those of a commercial SwIAV vaccine. The encapsulated mChit-NP vaccine induced high virus-specific neutralizing antibody and robust cellular immune responses, while the adsorbed vaccine elicited specific high IgG and hemagglutinin inhibition antibodies. Importantly, both the mChit-NP vaccines reduced challenge heterologous viral replication in the nasal cavity higher than commercial swine influenza vaccine. In summary, a novel intranasal mChit-NP vaccine platform activated both the arms of the immune system and is a significant advancement in swine influenza vaccine design, demonstrating its potential effectiveness for pig immunization.

## 1. Introduction

Swine influenza, caused by various influenza A virus (IAV) subtypes such as H1N1, H1N2, and H3N2, remains a significant global infectious respiratory threat, marked by epidemic outbreaks across diverse regions [1,2]. The consequential economic impact on hog farmers is multifaceted, with SwIAV infections leading to diminished productivity, prolonged time to slaughter, and a reduced number of piglets per sow in influenza-endemic herds [3].

Existing commercial vaccines against SwIAV primarily consist of inactivated, adjuvanted, multivalent whole virus formulations, targeting prevalent strains like H1N1, H1N2, and H3N2 in Europe and North America [4]. However, these vaccines, administered intramuscularly, exhibit limitations in effectively inducing immunity at the virus entry site in the respiratory tract, particularly in the upper airways [5].

Intranasal vaccination emerges as a promising avenue, capable of stimulating both mucosal and systemic immunity, potentially offering superior protection against influenza compared to parenteral administration [6,7]. The nasal mucosa, an advantageous site for protein antigen delivery due to its low proteolytic activity and considerable absorption capacity [8], sets the stage for innovative approaches. Mucoadhesive polymeric particles, including positively charged nanoparticles (NPs), demonstrate properties that overcome mucociliary clearance by attaching to negatively charged mucus on epithelial surfaces [9], ensuring protection for the antigen against degradation and rapid mucociliary clearance [10,11].

NP-based SwIAV vaccines utilizing whole inactivated virus antigen, alongside potent adjuvants, hold a strategic advantage in eliciting robust humoral and cellular immune responses at both mucosal sites within the respiratory tract and systemically. Chitosan (Chit) is a well-known polymer used in developing mucoadhesive NPs for vaccine delivery platforms. In the acidic environment of the nasal mucosa, the positively charged amine groups (NH₃⁺) of Chit interact electrostatically with the negatively charged mucus and nasal epithelial cells, providing mucoadhesive properties [12]. Our prior research demonstrated the effectiveness of Chit-NPs as an intranasal and oral vaccine delivery carrier for poultry and swine vaccines in terms of eliciting cross-presentation of antigens and robust cellular immune responses [13,14,15]. Notably, mChit-NPs were synthesized by conjugating native chitosan with the mannose adjuvant molecule. These mChit-NPs demonstrated enhanced adjuvant properties, effectively stimulating immune responses against both homologous and heterologous influenza viruses in pigs, particularly when delivering the intranasally administered monovalent H1N2 SwIAV antigen, compared to conventional chitosan NPs used in a standard formulation [15]. In these studies, the active components of the vaccine were delivered encapsulated into mChit-NPs. Notably, the surface adsorption of an antigen–adjuvant complex onto mChit-NPs, particularly in the context of mucosal vaccine studies, remains unexplored.

A STING-agonist ADU-S100 (ADU-100), also known as the stimulator of interferon genes (STING) agonist, emerges as a promising adjuvant for intranasal vaccine development. It stimulates STING as an intracellular checkpoint for gene transcription involved in many host defense mechanisms. This activation leads to the proliferation and maturation of immune cells such as lymphocytes and antigen-presenting cells, stimulating the nuclear factor-ĸB (NF-κB) and interferon regulatory transcription factor 3 (IRF3) signaling pathways [16]. The STING agonist ADU-S100 (ADU-S100) further augments its potential as a vaccine adjuvant by inducing interferon-gamma (IFN-γ) secretion in co-cultures [17]. Encouragingly, the intranasal administration of the H1N2 SwIAV antigen with ADU-S100 demonstrated robust immune responses against both homologous and heterologous influenza viruses in pigs, surpassing the efficacy of intranasal vaccination without ADU-S100 [18]. Despite the individual advantageous properties of mChit-NPs and ADU-S100 for intranasal SwIAV vaccine development, their combined utilization has not been thoroughly explored.

Therefore, aim of this study was to demonstrate humoral and cellular immune responses in a mChit-NPs vaccine containing inactivated OH10-H1N2 SwIAV [A/swine/Ohio/FAH10-1/2010 (H1N2)] antigen and ADU-S100 adjuvant, delivered intranasally in pigs. The vaccines’ efficacy was evaluated using a challenge with a heterologous pandemic CA09-H1N1 influenza A virus [A/California/04/2009 (H1N1)]. To empirically assess this hypothesis, the vaccine antigen–adjuvant complex was either encapsulated or surface adsorbed on pre-formed mChit-NPs [12,19].

## 2. Materials and Methods

### 2.1. Cells and Viruses

The vaccine virus used was swine influenza A virus OH10-H1N2 [A/swine/Ohio/FAH10-1/2010 (H1N2)] [20] and the challenge virus was influenza A virus CA09-H1N1 [A/California/04/2009 (H1N1)] [21]. Both viruses were grown in Madin–Darby canine kidney epithelial cells (MDCK, CRL-2285, ATCC, Manassas, VA, USA) [14]. In brief, MDCK cells were grown in Dulbecco’s Modified Eagle medium (DMEM) containing 10% heat inactivated fetal bovine serum (FBS) (Bio-techne, Minneapolis, MN, USA), antibiotic–antimycotic (Gibco, Thermo Fisher, Waltham, MA, USA), and 1 M HEPES in normal saline. Cells were infected with H1N2 IAV strain at 0.001 multiplicity of infection (MOI) and the cell culture supernatant was concentrated/purified using a Pellicon-2 mini cassette (Millipore, Burlington, MA, USA). Ultracentrifugal purification of the antigen was performed using a sucrose cushion method for 4 h at 25,000× *g* with no break. The viral pellet was suspended in phosphate-buffered saline (PBS) treated with protease inhibitors (Sigma, St. Louis, MO, USA). The virus inactivation was conducted using binary ethyleneimine 10 mM (Sigma) for 6 h at 37 °C, with subsequent incubation for 2 h at 37 °C with 10 mM sodium thiosulphate. Virus inactivation was confirmed in MDCK cell culture, and protein concentration was determined using BCA protein assay kit (Thermo Fisher) and the influenza hemagglutination (HA) units were also determined.

### 2.2. Vaccine Formulations

Bovine serum albumin (BSA) was utilized as a model protein to encapsulate or adsorb on mChit-NPs to evaluate in vitro cytotoxicity on pig peripheral blood mononuclear cells (PBMCs). The physiochemical properties of NPs were confirmed in Zetasizer instrument (Malvern Panalytical Ltd., Malvern, UK) for particle size, zeta potential, polydispersity index (PDI), and loading efficiency, as well as the loading capacity of both the antigen and adjuvant [19]. The whole inactivated SwIAV-loaded mChit-NPs were prepared with both the surface adsorption method and encapsulation method.

For the mChit-NPs surface adsorbed vaccine, the inactivated whole SwIAV H1N2 was surface adsorbed on the mChit-NPs. The chitosan NPs were prepared using the ionotropic gelation method, which relies on the ionic charge interaction, possessing a high positive charge, and sodium tripolyphosphate (TPP), possessing a negative charge [19]. Briefly, chitosan was dissolved in 1% acetic acid solution to achieve pH 5.5 and to obtain a concentration of 3 mg/mL under gentle stirring at room temperature, then chemically conjugated with mannose to obtain mChit-NPs. At the same time, the antigen and adjuvant mixture was prepared by mixing the inactivated SwIAV antigen and synthetic ADU-S100 (MIW815, Indianapolis, IN, USA) at equal ratios (1.25 mg: 1.25 mg) into a MOPS buffer at pH 7.4. The mixture was mixed with mChit-NPs with the mass ratio of 1:9 and gently stirred for 60 min to allow protein antigen adsorption on the surface of the mChit-NPs, resulting in mChit-SwIAV + ADU-S100 surface adsorbed NPs (mChit-SwIAV + S100-**s**NPs). The NPs were centrifuged for 40 min at 14,000× *g*, and the pellet was resuspended in 1× PBS pH 5.5 and kept at 4 °C. The supernatant was estimated to determine the percentage loading efficiency (LE) and loading capacity (LC) of the surface adsorbed OH10-H1N2 SwIAV antigen and the ADU-S100 adjuvant into mChit-NPs with a micro BCA protein assay kit and nanodrop spectrophotometer at 260 nm [22].

For the mChit-NPs encapsulated vaccine, we followed the method described previously [15]. Briefly, the mixture of inactivated SwIAV and ADU-S100 was mixed into a chitosan solution of 1 mg/mL. The TPP was added for cross-linking to generate mChit-SwIAV + ADU-S100-encapsulated NPs (mChit-SwIAV + S100-**e**NPs) vaccine. The NPs were collected by centrifugation and a supernatant was used for LE and LC evaluation. The antigen loading in mChit-SwIAV + S100-eNP was estimated.

Both the mChit-SwIAV + S100-sNPs and mChit-SwIAV + S100-eNPs were freshly prepared for physical characterization with a Zetasizer to measure NP size distribution and zeta potential (Malvern Panalytical Ltd., Malvern, UK), then diluted in 1× DMEM prior to vaccination of the pigs.

### 2.3. Immunizations and Challenge Studies in Pigs

Caesarian-derived colostrum-deprived specific pathogen-free (SPF) large White Duroc crossbred piglets were reared in a BSL-2 animal facility. Pigs aged 4 weeks were randomized into five groups, with *n* = 6 per group. Vaccine groups included: (1) mock-unvaccinated, no challenge infection; (2) unvaccinated mock and infectious virus challenge; (3) mChit-SwIAV + S100-eNPs + challenge; (4) mChit-SwIAV + S100-sNPs + challenge; (5) commercial vaccine (FluSure XP^TM^, Zoetis, Parsippany-Troy Hills, NJ, USA) + challenge. Animals were mock-vaccinated with PBS or vaccinated intranasally with 1 mL per dose (0.5 mL/nostril) of one of the mChit-SwIAV + S100-NPs in a prime-boost regime at three weeks apart, using a spray mist vaccinator (Prima Tech USA, Kenansville, NC, USA). Each intranasal vaccine dose contained 250 µg OH10-H1N2 antigen (~10,000 HA units) and 150 µg ADU-S100 adjuvant. The vaccine control group was intramuscularly injected with a commercial multivalent inactivated SwIAV vaccine (FluSure XP^TM^; Zoetis, USA), as per the manufacturer’s instructions, which contains four influenza isolates: [A/Swine/North Carolina/031/05 (H1N1)], [A/Swine/Iowa/110600/00 (H1N1)], [A/Swine/Oklahoma/0726H/2008 (H1N2)], [A/Swine/Missouri/069/05 (H3N2)]. All animal groups, except the mock group, were challenged with influenza A virus, 2009 pandemic CA09-H1N1 (A/California/04/2009), 2 × 10^7^ TCID_50_/mL in 1 mL DMEM, administered with 0.5 mL by intranasal and 0.5 mL by intratracheal routes at two weeks after the booster vaccination. Pigs were monitored daily for clinical signs, measuring body temperatures regularly until 6 days post-infection (DPI). Blood samples and nasal swabs were collected before immunization and at DPI-2, -4, and -6. Pigs were euthanized at DPI-6 and blood was collected for serum and extraction of PBMCs, nasal swabs, lung tissues for lung lysate, and tracheobronchial lymph nodes (TBLNs) to isolate mononuclear cells (MNCs) and bronchioalveolar lavage (BAL) fluid.

All experiments in this animal study were performed in accordance with the Guide for the Care and Use of Agricultural Animals in Agricultural Research and Teaching, the National Research Council’s Guide for the Care and Use of Laboratory Animals, Public Health Service Policy, and USDA Regulations. The animal study protocol was approved by the Institutional Review Board (Ethics Committee) of Ohio State University (protocol # 2014A00000099-R3).

### 2.4. Hemagglutination Inhibition Assay

The hemagglutination inhibition (HI) titer specific to the 2009 pandemic CA09-H1N1 antigen in sera and BAL fluid obtained at DPI-6 was determined as described previously [23]. Briefly, heat-inactivated and serially diluted pig samples were added into the antigen containing 8 HAU/50 µL and incubated for 1 h at 37 °C. HI titers were calculated as the reciprocal of the highest sample dilution that gave complete inhibition. Comparisons between animal groups was performed by using log_2_-transformed values.

### 2.5. Antibody ELISA

Enzyme-linked immunosorbent assay (ELISA) was performed using the influenza antigens CA09-H1N1 heterologous challenge strain, OH10-H1N2 homologous vaccine strain, and OH4-H3N2 heterosubtypic virus for measuring the secretory IgA and IgG antibodies in serum, nasal swab specimens, BAL samples, and lung lysate. ELISA plates (Greiner bio-one, Monroe, NC, USA) were coated in duplicate with inactivated and pre-titrated CA09-H1N1 at 8 µg/mL, OH10-H1N2, and OH4-H3N2 at 4 µg/mL antigen in 50 µL coating buffer (50 mM carbonate–bicarbonate, pH 9.6) per well overnight at 4 °C. The plates were washed three times with PBS-T (PBS with 0.05% Tween-20) and free binding sites were blocked with 5% non-fat dry milk in 50 µL PBS-T per well for 1 h at room temperature (RT). Samples of serum, nasal swab specimen, BAL fluid, and lung lysate were serially diluted in 2.5% dry milk in PBS-T and incubated overnight at 4 °C. Plates were washed three times with PBS-T, incubated with 50 µL/well HRP-coupled goat anti-pig IgA (dilution 1:2000, Bethyl Laboratories Inc., Montgomery, TX, USA), or peroxidase labeled goat anti-swine IgG (H  +  L) (dilution 1:8000) (Jackson ImmunoResearch Laboratories Inc., West Grove, PA, USA) detection antibodies in 2.5% dry milk in PBS-T for 2 h at RT. After three washings with PBS-T, 50 µL/well peroxidase substrate solution B and 3,3′,5,5′-Tetramethylbenzidine (Thermo Fisher Scientific, USA) were added in a 1:1 ratio. The plates were developed in the dark and the reaction was stopped by adding 50 µL/well of 1 M phosphoric acid, the signal was then measured on a microplate reader at 450 nm (Spectramax, Molecular Devices, San Jose, CA, USA), and the final optical density (OD) was obtained by subtracting the average of the duplicate test sample readings from the average blank.

### 2.6. Avidity of SwIAV-Specific Antibodies

The determination of IgG antibody avidity in vaccine groups for target CA09-H1N1 antigen was performed using ELISA and dilutions of samples including serum (1:500), BAL fluid and lung lysate (1:50), and nasal specimens (1:4), quantified in the presence of chaotropic agent ammonium thiocyanate (NH_4_SCN) [24,25]. In brief, following the steps completed as described above for the antibody ELISA, the plates were washed three times with PBS-T after overnight incubation of experimental samples at 4 °C, then kept for 15 min at RT with NH_4_SCN (Acros Organics, Fair Lawn, NJ, USA) diluted in PBS-T at decreasing concentrations from 5 M to 0 mM. For the control, another set of samples were treated with PBS-T at 0 M of NH_4_SCN. Plates were washed with PBS-T, then incubated with secondary antibodies to determine the antigen and antibody reactivity by measuring OD values at 450 nm using the plate reader. Relative binding of anti-CA09-H1N1 antibody was calculated by subtracting the average corresponding OD values of diluted NH_4_SCN at various molar concentrations from the average OD 450 value without NH_4_SCN (0 mM) treatment, then by plotting non-linear regression against NH_4_SCN concentrations. The antibody avidity index (AI) was calculated by dividing mean OD readings at 1.25 M NH_4_SCN by 0 M readings and multiplying by 100 (OD 1.25 M/OD 0 M) × 100.

### 2.7. Proliferation Assay

In vitro proliferative response was measured by tetrazolium uptake in proliferating PBMCs and TBLN MNCs [14]. In brief, 1 × 10^6^ cells were cultured in RPMI medium with 10% FBS in a flat bottom 24-well plate in duplicates and kept for 68 h at 37 °C after the stimulation or non-stimulation with CA09-H1N1 virus at 0.1 MOI cultured with 2 ng/mL of recombinant porcine IL-2 (Kingfisher Biotech, Saint Paul, MN, USA). After incubation cells were treated with MTS and PMS solution (Promega, Madison, WI, USA) with 20 µL/well and incubated for 4 h at 37 °C in the dark, the absorbance spectra at OD 490 nm was measured with microplate reader. Corrected absorbance was calculated by subtracting the average of the blank absorbance values from all other experiential OD values. The stimulation index is defined as the quotient obtained between the mean OD of the CA09-H1N1-stimulated cells and the respective OD mean of the unstimulated cells.

### 2.8. Flow Cytometry

Single cell suspension of isolated PBMCs and TBLN MNCs [26] were cultured in complete RPMI medium containing 10% FBS and 2 ng/mL of recombinant porcine IL-2 (Kingfisher Biotech), then in vitro stimulated with 0.1 MOI CA09-H1N1 virus for 48 h at 37 °C. The BD GolgiPlug Protein Transport Inhibitor (BD Bioscience, Franklin Lakes, NJ, USA) was added to each well for the last 6 h of incubation and, after washing, cells were blocked with 1% normal rabbit serum for 30 min at 4 °C. Subsequent surface and intracellular immunolabeling were performed using antibodies for myeloid cells, for IL-17A positive T-helper/memory cells, and for gamma interferon (IFN-γ) positive cytotoxic T lymphocytes (CTL) (Supplementary Data Table S1). The flow cytometry results were analyzed using FlowJo^TM^ v10.8 Software (BD Life Sciences, Franklin Lakes, NJ, USA).

### 2.9. Virus Titration and Neutralization Assay

The challenge virus titer in nasal swab specimens collected at DPIs-2, -4, and -6 were assayed in triplicates inoculating MDCK cell monolayers seeded for 24 h prior with 2 × 10^4^/well in DMEM supplemented with 10% FBS, 5% antibiotic–antimycotic, and 10% 1 M HEPES (Thermo Fisher) in flat bottom 96-well plates at 37 °C in 5% CO_2_. Nasal swab samples serially diluted in DMEM (with no added FBS) at 100 µL/well were inoculated onto the cells’ monolayer and incubated at 37 °C in a 5% CO_2_ for 1.5 h before the addition of 100 µL/well DMEM containing 2.0 µg/mL TPCK-treated trypsin (Sigma, USA) and kept for 40 h at 37 °C in a 5% CO_2_. Cells were fixed with 80% acetone in PBS and the presence of CA09-H1N1 infected cells was determined adding influenza A virus monoclonal antibodies (CalBioreagents, Foster City, CA, USA) for 2 h, with subsequent addition of secondary goat anti-mouse IgG (H + L) conjugated with AF488 (Life Technologies, Eugene, OR, USA). Influenza viral foci were read in each well at triplicate sample dilution using a fluorescent microscope (Olympus, Huntington, NY, USA), and the virus titer TCID_50_/mL was calculated with the Reed–Muench method transformed to log_10_ basis following previous study [26].

Virus neutralizing antibody titers were determined as follows: sera collected at DPI-6 were heat inactivated at 56 °C for 30 min. Serial dilutions of 100 µL/well sera were incubated with 100 µL/well of CA09-H1N1 virus at 50 TCID_50_/well for 1.5 h at 37 °C. The sample and virus mix of 100 µL/well was transferred onto pre-formed MDCK cells’ monolayer and incubated for 1.5 h at 37 °C in 5% CO_2_ incubator before the addition of 100 µL/well 1× DMEM containing 2.0 µg/mL TPCK-treated trypsin, and further incubated for 40 h. Subsequently, similar procedures to those described above were performed for virus titration. The neutralizing activities in samples were measured as the reciprocal of the highest sample dilution inhibiting 100% virus infection of MDCK cells [27].

### 2.10. Influenza HA Multiple Sequence Alignments

We performed multiple sequence alignments using the BLAST server [28] for influenza A virus HA gene sequences’ similarity between vaccine antigen strain—[A/swine/Ohio/FAH10-1/2010 (H1N2)] GenBank accession: HQ833566, challenge virus—[A/California/04/2009 (H1N1)] GenBank accession: EU409948, and heterosubtypic strain [A/turkey/Ohio/313053/2004 (H3N2)] GenBank accession: EU735818, as well as commercial vaccine strains—[A/swine/NC/00573/2005 (H1N1)] Genbank accession: FJ638306.1, [A/swine/Oklahoma/032726/2008 (H1N2)], Genbank accession: CY045519.1, [A/swine/Minnesota/SG1236/2005 (H3N2)], Genbank accession: CY157855.1 [A/swine/Mexico/Mex52/2010 (H1N1)], Genbank accession: CY122357. The latter two sequences (Minnesota/2005 and Mexico/2010) are a substitute sequence 98–99% homologous to the commercial vaccine original strains provided by Zoetis, USA on request.

### 2.11. Statistical Analysis

Statistical differences between multi-group comparisons were performed using one-way ANOVA or two-way ANOVA followed by Tukey’s multiple comparison test in Prism 10 (GraphPad Software, Inc., San Diego, CA, USA). The group data are presented as the mean ± SEM of five to six pigs, with statistical significance of *p* < 0.05. A box-and-whisker plot shows the interquartile ranges, and horizontal lines show the group median.

## 3. Results

### 3.1. Vaccine Formulations

Bovine serum albumin (BSA) was utilized as a model protein to evaluate in vitro cytotoxicity of mChit-NPs encapsulated or surface adsorbed with BSA tested on pig PBMCs. The physiochemical properties of NPs were confirmed in Zetasizer instrument (Malvern) for particle size distribution, zeta potential, and PDI. Furthermore, we assessed the NPs’ loading efficiency, as well as the loading capacity of both the antigen and the adjuvant [19]. It was found that both the antigen loading methods, encapsulation and surface adsorption, slightly decreased the NPs’ positive charge (zeta potential) and increased the particle size and PDI values as the mass ratio of antigen (Ag) to NP rises compared to the blank no protein mChit-NPs (Table 1).

However, the 1:9 mass ratio of Ag to NPs presented a homogeneity in size, with PDI < 0.5 in both loading methods. Moreover, at a 1:9 mass ratio, both the mChit-NPs exhibited nano-size particles (333–348 nm) with a positive surface charge (+17–+19 mV) (Table 1). The PDI was 0.3–0.4, which was lower than 0.5, indicating homogeneous distribution of the particles [12]. These NPs are suitable for suspending as individual particles, due to their high positive charge. These values are the minimal requirements of chitosan-based NPs for superior physical stability. Therefore, both loading methods of NPs at 1:9 Ag/NPs are optimized and selected for preparation of NP vaccines for studies in pigs.

**Figure 1:**
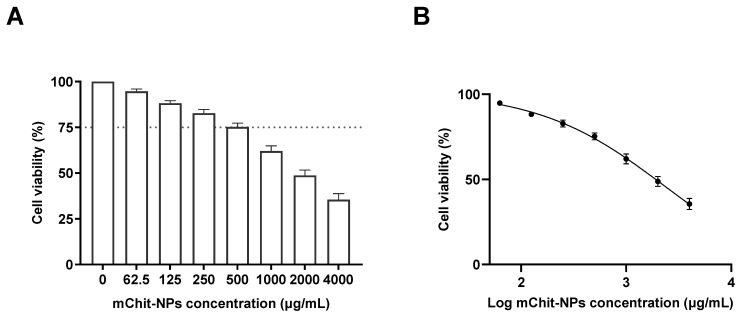
Evaluation of cytotoxicity of mChit-NPs on pig PBMCs. Different concentrations of mChit-NPs (µg/mL) encapsulated with BSA were assessed for toxicity on PBMCs using the MTS assay. The results are presented as follows: (**A**) linear bar graph; the dotted line represents 75% cell viability at a concentration of 500 µg/mL mChit-NPs. (**B**) Exponential curve: utilizing non-linear regression analysis on PBMCs viability data enabled the extrapolation of the 50% inhibitory concentration (IC) 50 value at 2200 µg/mL. Mean ± SEM obtained from a single experiment (*n* = 4).

The cytotoxicity of mChit-NPs was evaluated on freshly extracted PBMCs by determining formazan product residues expressed as a percentage of detected viable cells on a plate reader using the MTS cytotoxicity assay kit. The concentration of mChit-NPs below 500 µg/mL exhibited as biocompatible, with over 75% viable PBMCs (Figure 1A,B).

As shown previously, the concentration of chitosan NPs biocompatible on cells is 70% [29]. Based on the calculated 50% inhibitory concentration (IC50), 2200 µg/mL of mChit-NPs had decreased cell viability. Moreover, chitosan NPs had IC50 on PBMCs at 720 µg/mL and the relationship between chitosan NP concentration and cell viability is shown in a non-linear regression analysis [29]. According to this study and its findings and further explanations of chitosan on cell viability, mannose–chitosan NPs exhibited lower cytotoxicity than chitosan NPs on HepG-2 and SMMC-7721 cells [30]. In our vaccine study, mChit-NPs were loaded with whole inactivated OH10-H1N2 SwIAV and ADU-S100 adjuvant, using both encapsulation and surface adsorption methods. The characterization of mChit-NPs loaded with the whole inactivated OH10-H1N2 antigen via both surface adsorption and encapsulation methods created mChit-NPs that exhibited a homogeneous nano-sized distribution (PDI < 0.5) with a positive charge (Table 2). The size distribution profiles of mChit-NPs loaded with the OH10-H1N2 antigen is provided in Supplementary data Figure S1. After loading the OH10-H1N2 antigen or adjuvant using both the methods, the size increased and the zeta potential decreased, indicating the successful loading of antigen–adjuvant in mChit-NPs. Due to electrical properties, protein antigens that contain carboxyl groups (COO^−^) with a negative charge interact with the positively charged amine groups of Chit. As a result, the surface adsorption method led to an increased size and decreased zeta potential of mChit-NPs due to antigen loading on the surface. In the encapsulation method, the antigen was mixed with mChit before preparing mChit-NPs using an ionic gelation technique involving interaction between the positive charge of Chit with the negative charge of TPP, resulting in the antigen encapsulated in the matrix/core of mChit-NPs. Therefore, mChit-NPs loaded with the OH10-H1N2 antigen via the encapsulation method had a slightly smaller nano-size and slightly higher zeta potential compared to NPs loaded via surface adsorption; 336.5 ± 33.7 nm with a zeta potential of 18.6 ± 1.8 mV versus 396.5 ± 44.1 nm with a zeta potential of 17.4 ± 1.7 mV, respectively (Table 2). Importantly, the physical nature of mChit-NPs loaded with either of the proteins, OH10-H1N2 antigen or BSA, were not different, indicating that BSA is a suitable protein antigen model for mChit-NP vaccine formulation as previously described [12,19]. The loading efficiency (LE) and loading capacity (LC) data show that both the encapsulated and surface adsorbed mChit-NPs had similar levels of LE and LC values; 65–71% and 3.4–3.7% for antigen loading and 45–51% and 3.3–3.7% for adjuvant loading, respectively (Table 3).

The stock vaccine antigen protein concentration was 4.5 mg/mL and the functional antigen it contained was 204,800 HA units per 1 mL. For intranasal vaccination, each dose of vaccine formulation contained 250 µg total protein which had ~10,000 HA units of antigen and 150 µg ADU-S100 adjuvant in 1 mL dose.

### 3.2. Hemagglutinin Gene Nucleotide Identity Assessment

**Figure 2:**
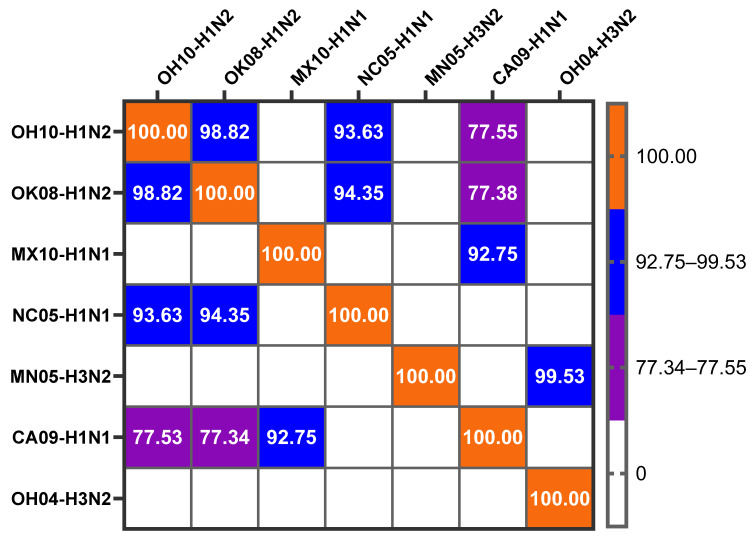
Heatmap illustrating percentage nucleotide identity of HA gene of different vaccine SwIAVs compared to 2009 pandemic CA09-H1N1 challenge virus. Multiple sequence alignments of influenza A virus HA gene sequences were conducted using the BLAST server. The main comparisons related to our study include analysis of intranasal vaccine antigen strain OH10-H1N2 with challenge virus CA09-H1N1 and the commercial vaccine virus strains NC05-H1N1, OK08-H1N2, MN05-H3N2, and MX10-H1N1, with challenge virus CA09-H1N1.

Influenza surface HA glycoprotein is the major target for inducing humoral immune responses, and its subunit HA2 glycopolypeptide molecule remains highly conserved among other subtypes, with 51–80% homology [31,32]. We assessed HA gene similarities between our monovalent mChit-SwAIV-NP vaccine virus and a commercial multivalent vaccine containing multiple SwAIV subtypes by retrieving data of isolates from the GenBank public database [33], then using the BLAST server [28] to align them against the challenge virus CA09-H1N1. The commercial vaccine SwIAV HA MX10 (H1N1) and OK08 (H1N2) genes are highly conserved, maintaining 77.34% and 92.75% sequence identity, respectively (Figure 2).

The nucleotide similarity of our intranasal vaccine antigen OH10-H1N2 retains 77.53% sequence identity with that of the CA09-H1N1 virus HA gene. Based on the HA gene nucleotide sequence identity between OH10-H1N2 and CA09-H1N1 subtypes, these two viruses are heterologous to each other, while the commercial vaccine viruses are homologous with challenge CA09-H1N1 virus with closer HA gene similarity. Therefore, if the mChit-SwAIV-NP vaccine induces superior adaptive immunity following prime-boost vaccination, then the vaccine is considered cross-protective.

### 3.3. Cross-Protective Efficacy against Upper-Respiratory Tract Influenza Infection

**Figure 3:**
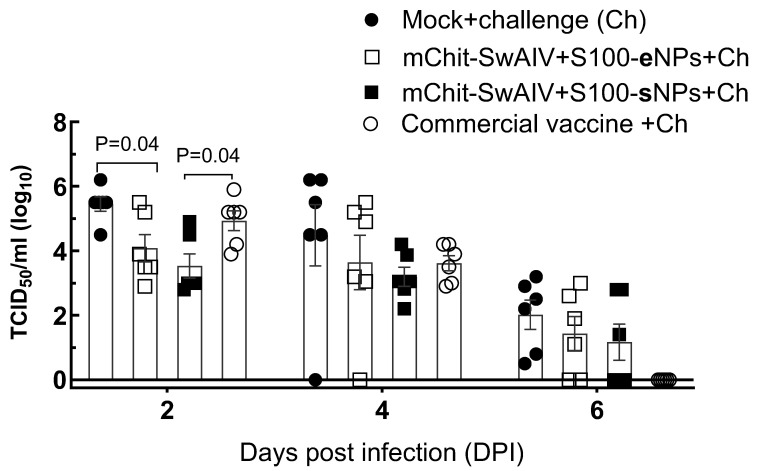
Efficacy of both the mChit-SwAIV + S100-NP vaccines against viral replication in the upper respiratory tract. Nasal swab specimen collected at DPIs-2, -4 and -6 were subjected to influenza A virus titration by estimating the 50% tissue culture infectious dose (TCID_50_). Data are presented as TCID_50_ log_10_ viral loads, individual symbols indicate single pig value, and each bar is the mean ± SEM of six pigs in each group. A line drawn above the titer value ‘0’ indicates the limit of virus detection. The *p* values between groups (*p* < 0.05) were determined by one-way ANOVA with Tukey’s multiple comparisons post-hoc test.

The efficacy of the mChit-SwAIV-NP intranasal monovalent inactivated whole OH10-H1N2 strain vaccine was compared to the commercial inactivated multivalent intramuscular vaccine containing four SwIAV strains, two H1N1 and one of each H1N2 and H3N2 strains. The immunization dose remained constant across two types of mChit-NPs vaccine groups and for commercial vaccine immunization, the manufacturers protocol was followed. Two weeks after prime-boost immunization, all pig groups, except the mock group, were challenged with the 2009 pandemic CA09-H1N1 virus. Following the virus challenge, pigs did not show any clinical signs of disease. We assessed viral loads at DPIs-2, -4 and -6 in nasal swabs (NS). At DPI-2 the highest viral load was detected in the commercial vaccine group compared to the NP-based vaccine groups (Figure 3).

In both the types of mChit-NPs based SwIAV vaccine formulations administered vaccine groups detected lower levels of virus (*p* < 0.05) compared to control challenge animals. At DPI-4, one of six animals in the mChit-SwIAV + S100-eNPs (Ag encapsulated) group and four of six animals in the mChit-SwIAV + S100-sNPs (Ag surface adsorbed) group had numerically lower infectious virus load. In the mock control and commercial vaccine groups, much higher virus loads were detected (average log_10_ 3.7). Finally, by day six, although infectious virus loads in both the intranasal vaccine groups were substantially cleared, 2–3 animals still had a detectable virus, while in the commercial vaccine group, none of the six animals had detectable viral load by day six. Overall, the infectious virus load was reduced by around 2 log_10_ in the nasal passage of both the encapsulation and surface adsorbed mannose–chitosan NPs vaccinations at DPI-2, with the latter performing relatively better within the first four days post-infection.

### 3.4. Hemagglutinin Inhibition and Virus Neutralizing Antibody Titers in Vaccinated Pigs

**Figure 4:**
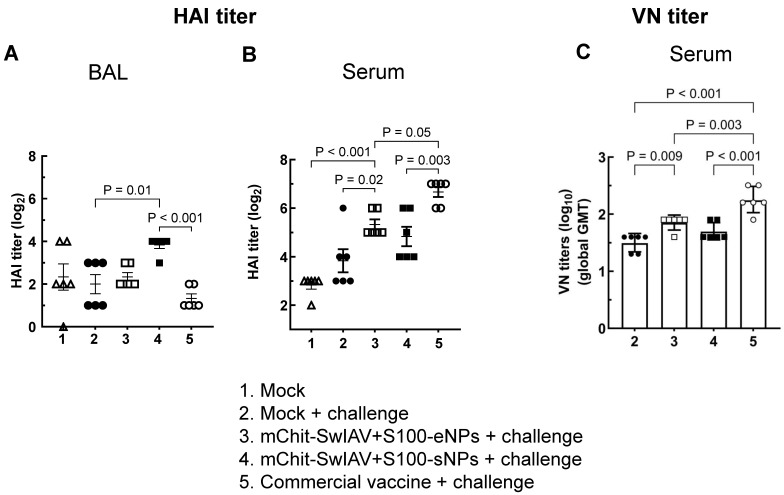
Hemagglutination inhibition (HAI) and virus neutralization (VN) antibody titers in pigs immunized with mChit-SwAIV + S100-NP vaccines and challenged with a heterologous influenza A virus. Pigs were prime-boost vaccinated intranasally with either encapsulated or surface adsorbed mannose–chitosan NPs vaccine or intramuscularly with a commercial vaccine. (**A**) BAL fluid, (**B**) serum antibody HAI endpoint titers, and (**C**) VN titers in serum collected at DPI-6 were assessed against the challenge CA09-H1N1 virus. Each marking represents the titer of an individual pig in a group (*n* = 6). HAI titers were transformed to log_2_ values and error bars indicate means ± SEM. VN titer is the reciprocal endpoint titer transformed to log_10_ value and plotted as a geometric mean titer with SD (GMT) from triplicate wells. The *p* values between groups (*p* < 0.05) were determined by one-way ANOVA with Tukey’s multiple comparisons post-hoc test.

All immunized groups had virus specific antibodies at week six after prime-boost immunization and one-week post challenge against OH10-H1N2 virus Ag. The hemagglutinin inhibition antibody (HAI) titers and virus neutralizing activity against the challenge CA09-H1N1 virus were detected in all vaccine groups (Figure 4A–C). HAI titer in BAL fluid was significantly higher in the mChit-SwIAV + S100-sNPs vaccine group compared to the commercial vaccine and mock challenge groups (Figure 4A). The serum HAI titer in the commercial vaccine group (log_2_ mean = 6.67) was significantly higher than both the in both types of mChit-SwIAV + S100-NP vaccine groups (Figure 4B). The lowest HAI titer was detected among the vaccinates in the BAL fluid of commercial vaccine group (log_2_ mean = 1.33) (Figure 4A). The mChit-SwIAV + S100-eNPs (but not mChit-SwIAV + S100-sNPs) group and the commercial vaccine group elicited significantly higher virus neutralization titers in serum, compared to the mock challenge group (Figure 4C). These results indicate that intranasal vaccination with mChit-NPs elicits high HAI titers in the lungs, whereas the commercial SwIAV vaccine elicited elevated HAI as well as virus neutralizing activity in the serum.

### 3.5. Cross-Reactive Secretory (s) IgA and IgG antibodies Induced by mChit-NPs Vaccines

**Figure 5:**
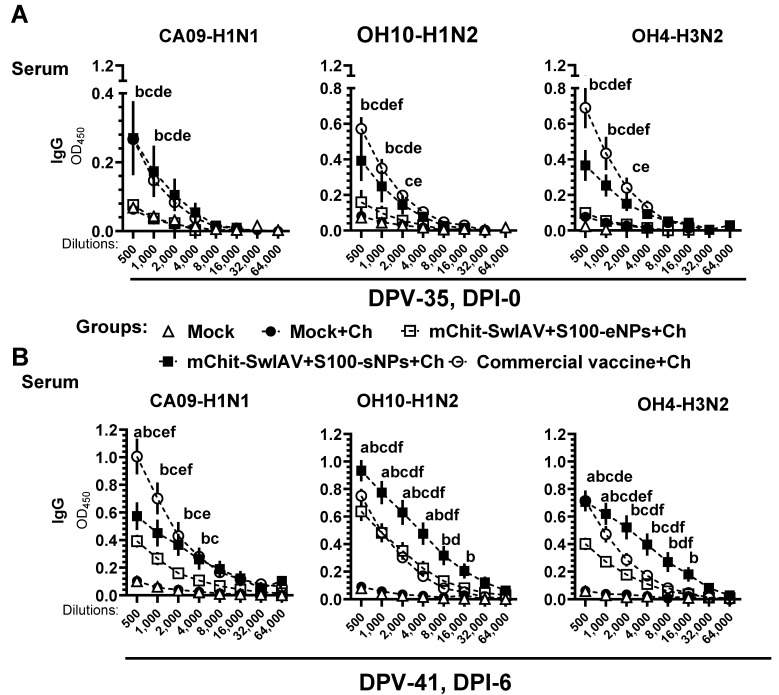
Influenza A virus specific IgG antibody responses in the serum of mChit-SwAIV + S100-NP vaccinated pigs challenged with a heterologous virus. Pigs were prime-boost vaccinated with either encapsulated or surface adsorbed mChit-SwAIV + S100-NPs vaccine intranasally, or intramuscularly with a commercial vaccine. Serum samples collected at (**A**) DPI-0 and (**B**) DPI-6 were assessed for specific IgG antibody responses against CA09-H1N1, OH10-H1N2, and OH4-H3N2 strains of viruses by ELISA. Each data point on the horizontal lines is the mean ± SEM values of 5–6 pigs. Alphabets above markings indicate significant difference between vaccine groups at a specific dilution such as: a—mock + challenge vs. mChit-SwAIV + S100-eNPs; b—mock + challenge vs. mChit-SwAIV + S100-sNPs; c—mock + challenge vs. commercial vaccine; d—mChit-SwAIV + S100-eNPs vs. mChit-SwAIV + S100-sNPs; e—SwAIV + S100-eNPs vs. commercial vaccine; f—mChit-SwAIV + S100-sNPs vs. commercial vaccine. The *p* values between groups (*p* < 0.05) were determined by two-way ANOVA with Tukey’s multiple comparisons post-hoc test.

We determined the levels of cross-reactive sIgA and IgG antibodies present in serum, BAL fluid, lung lysate, and nasal swab samples across diverse strains of SwIAV induced by mChit-NP-based inactivated OH10-H1N2 vaccine by ELISA using antigens derived from homologous OH10-H1N2, heterologous CA09-H1N1, and heterosubtypic OH04-H3N2 viruses. The specific IgG antibody titers in serum after prime-boost vaccination at DPI-0 against CA09-H1N1 antigen in mChit-SwIAV +S100-sNPs group had higher levels in comparison to its binding to OH10-H1N2 and OH4-H3N2 antigens when juxtaposed with the commercial vaccine (Figure 5A). The cross-reactive IgG response to heterologous and heterosubtypic antigens was increased by DPI-6. Indeed, mChit-SwIAV + S100-sNPs intranasally vaccinated pigs had higher titers of serum antibodies to OH10-H1N2 and OH4-H3N2 antigens compared to commercial intramuscular vaccinates (Figure 5B). These data show that intranasal vaccination, especially with mChit-SwIAV + S100-sNPs, stimulated enhanced cross-reactive IgG antibody responses in the serum of pigs.

**Figure 6:**
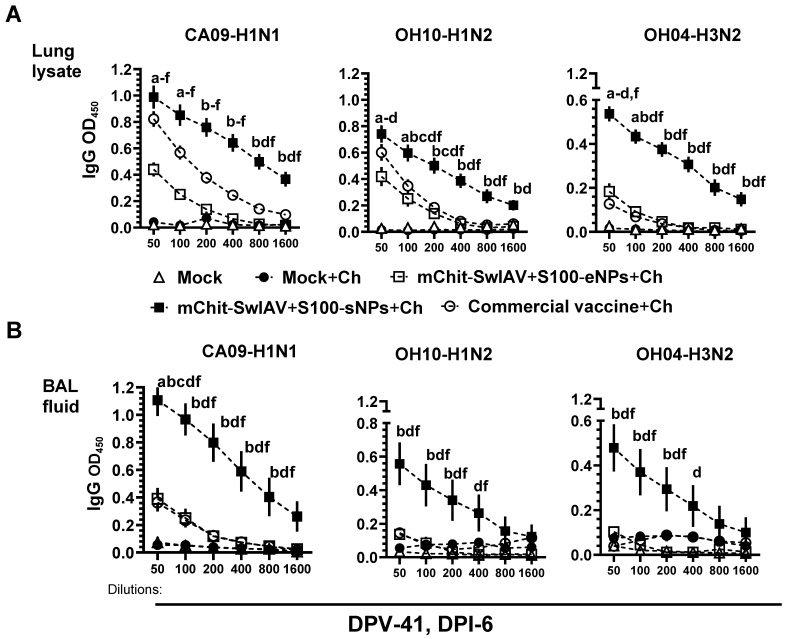
Influenza A virus specific IgG antibody responses in the lungs of mChit-SwAIV + S100-NP vaccinated pigs challenged with a heterologous virus. Pigs were prime-boost vaccinated with either encapsulated or surface adsorbed mChit-SwAIV + S100-NPs vaccine intranasally, or intramuscularly with a commercial vaccine. (**A**) Lung lysate and (**B**) BAL fluid specimenscollected at DPI-6 were assessed for specific IgG antibody responses against CA09-H1N1, OH10-H1N2 and OH4-H3N2 strain of viruses by ELISA. Each point on horizontal lines is the mean ± SEM values of 5–6 pigs. Alphabets above markings indicate significant difference between vaccine groups at a specific dilution such as: a—mock + challenge vs. mChit-SwAIV + S100-eNPs; b—mock + challenge vs. mChit-SwAIV + S100-sNPs; c—mock + challenge vs. commercial vaccine; d—mChit-SwAIV + S100-eNPs vs. mChit-SwAIV + S100-sNPs; e—SwAIV + S100-eNPs vs. commercial vaccine; f—mChit-SwAIV + S100-sNPs vs. commercial vaccine. The *p* values between groups (*p* < 0.05) were determined by two-way ANOVA with Tukey’s multiple comparisons post-hoc test.

In lung lysate and BAL fluid samples, the reactivity of specific IgG was predominantly higher against the homologous, heterologous, and heterosubtypic influenza virus antigens in mChit-SwIAV + S100-sNP vaccine group compared to the mChit-SwIAV + S100-eNP and commercial vaccine groups at DPI-6 (Figure 6A,B).

**Figure 7:**
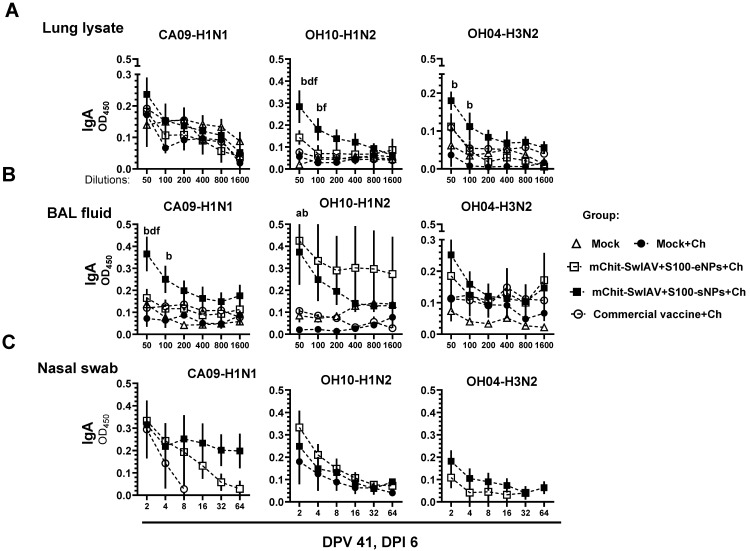
Influenza A virus specific sIgA antibody responses in the respiratory tract of mChit-SwAIV + S100-NPs vaccinated pigs challenged with a heterologous virus. Pigs were prime-boost vaccinated with either encapsulated or surface adsorbed mChit-SwAIV + S100-NPs vaccine intranasally, or intramuscularly with a commercial vaccine. (**A**) Lung lysate, (**B**) BAL fluid, and (**C**) Nasal swab specimenscollected at DPI-6 were assessed for specific sIgA antibody responses against CA09-H1N1, OH10-H1N2, and OH4-H3N2 strain of viruses by ELISA. Each point on horizontal lines is the mean ± SEM values of 5–6 pigs. Alphabets above markings indicate significant difference between vaccine groups at a specific dilution such as: a—mock + challenge vs. mChit-SwAIV + S100-eNPs; b—mock + challenge vs. mChit-SwAIV + S100-sNPs; d—mChit-SwAIV + S100-eNPs vs. mChit-SwAIV + S100-sNPs; f—mChit-SwAIV + S100-sNPs vs. commercial vaccine. The *p* values between groups (*p* < 0.05) were determined by two-way ANOVA with Tukey’s multiple comparisons post-hoc test.

The results of sIgA antibody reactivity in the upper (nasal swab) and lower (lung lysate and BAL fluid) respiratory tracts indicated higher titers against all three tested SwIAV antigens in both types of mChit-SwIV-NP vaccinates, with relatively higher levels in the mChit-SwIV-sNPs vaccinated group compared to the commercial vaccine group (Figure 7A–C). Overall, intranasal mChit-SwIAV + S100-sNP vaccination was able to stimulate robust cross-reactive mucosal sIgA responses in the respiratory tract of pigs.

### 3.6. Enhanced Heterologous Virus Specific IgG Antibody Avidity in BAL Fluid of mChit-SwIAV + S100-sNP Vaccinated Pigs

**Figure 8:**
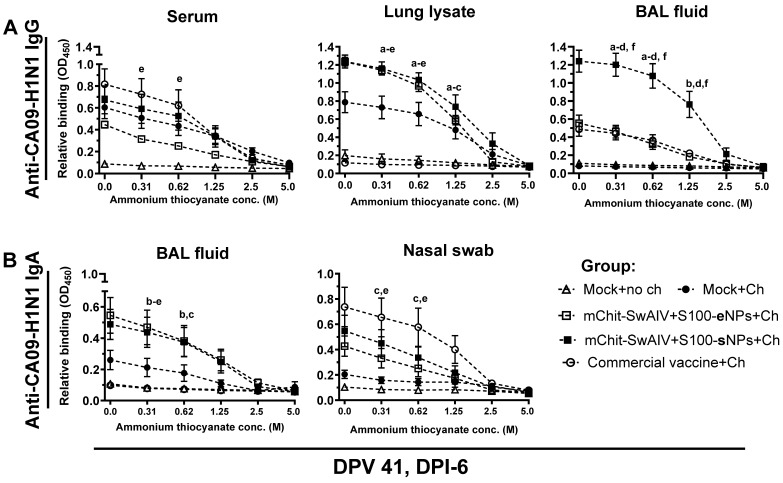
Avidity of cross-reactive influenza A virus specific IgG and sIgA antibody at various ammonium thiocyanate (NH_4_SCN) concentrations in the mChit-SwAIV + S100-NP vaccinated pigs challenged with a heterologous virus. Relative binding avidity of (**A**) IgG and (**B**) IgA to CA09-H1N1 antigen was assessed using a single test dilution of serum (IgG only), lung lysate, BAL fluid, and nasal swabs (sIgA only) in the absence or presence of NH_4_SCN at different concentrations. Each data point is the mean titer ± SEM from duplicate wells. Alphabets above markings indicate significant differences between vaccine groups at a specific dilution such as: a—mock + challenge vs. mChit-SwAIV + S100-eNPs; b—mock + challenge vs. mChit-SwAIV + S100-sNPs; c—mock + challenge vs. commercial vaccine; d—mChit-SwAIV + S100-eNPs vs. mChit-SwAIV + S100-sNPs; e—SwAIV + S100-eNPs vs. commercial vaccine; f—mChit-SwAIV + S100-sNPs vs. commercial vaccine. The *p* values between groups (*p* < 0.05) were determined by two-way ANOVA with Tukey’s multiple comparisons post-hoc test.

Avidity refers to the strength and stability of binding between an antibody and its antigen, and correlates with protective immunity induced by vaccines, especially protection at mucosal surfaces like the respiratory tract [24]. The dissociation of specific IgG antibodies from the cognate CA09-H1N1 antigen was assessed by subjecting serum, nasal swab, lung lysate, and BAL fluid samples to increasing concentrations of ammonium thiocyanate ranging from 0 to 5.0 M. The relative binding compared to the 0 M condition of ammonium thiocyanate was plotted using a non-linear regression model to identify the concentration at which dissociation occurred. At a chaotropic agent concentration of 1.25 M, antibodies were still bound to the antigen in all sample types. However, at concentrations above 1.25 M, the interaction between antibodies and antigen was dissociated (Figure 8).

**Figure 9:**
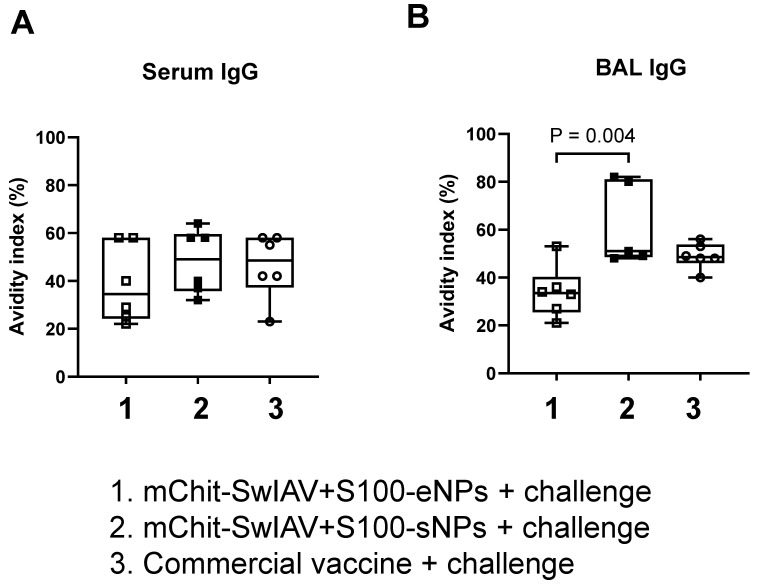
Avidity index of cross-reactive influenza A virus specific IgG antibody in mChit-SwAIV + S100-NPs vaccinated pigs challenged with a heterologous virus. The IgG avidity index in (**A**) serum and (**B**) BAL fluid was calculated using OD values obtained upon treatment, with single NH_4_SCN concentration at 1.25 M compared to untreated control samples. Box-and-whisker plot indicates interquartile ranges, horizontal lines show group median. The *p* values between groups (*p* < 0.05) determined by one-way ANOVA with Tukey’s multiple comparisons post-hoc test.

Following that, the avidity index (AI) for IgG in serum and BAL fluid was computed at an ammonium thiocyanate concentration of 1.25 M. In serum, the specific IgG avidity was comparable across all three vaccinated groups (Figure 9A). Conversely, in the mChit-SwIAV + S100-sNPs vaccinated group, a significantly higher avidity of virus-specific IgG in the BAL fluid, compared to that in mChit-SwIAV + S100-eNPs vaccinates, was observed (Figure 9B).

### 3.7. Enhanced Activation of Myeloid Cells in PBMCs and TBLN MNCs of mChit-NP Vaccinated Pigs

**Figure 10:**
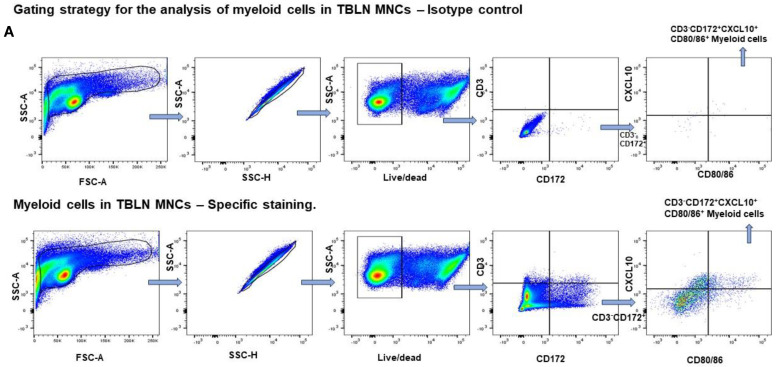

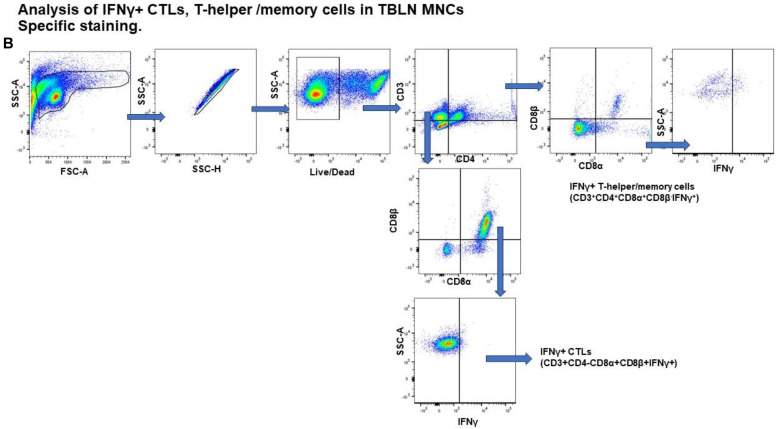

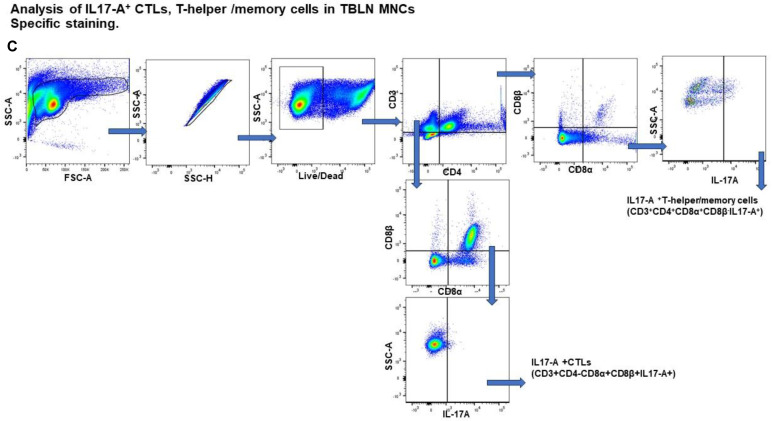
A representative gating strategy of pig TBLN MNCs by flow cytometry. (**A**) Top: Isotype control for myeloid cells; Bottom: specific antibody staining for myeloid cells; (**B**) IFNγ^+^ T-helper/memory cells and IFNγ^+^ cytotoxic T lymphocytes; (**C**) IL-17A^+^ T-helper/memory and IL-17A^+^ cytotoxic T lymphocytes. The flow cytometry results were analyzed using FlowJo Software.

It was determined whether mChit-NP vaccination could modulate early innate immune responses and induce trained immunity. Freshly isolated TBLN MNCs and PBMCs of pigs immunized and challenged with a heterologous virus were subjected to flow cytometry to phenotype the myeloid cells in vaccinates. For phenotyping, we gated for CD3^−^ (negative for T cells), CD172^+^ (myeloid marker), CXCL10^+^ (inflammatory chemokine), and CD80/86^+^ (co-stimulatory molecule) (Figure 10A). For phenotyping of T-helper/memory and cytotoxic T lymphocytes (CTLs), we used the cells gated for CD3^+^ phenotype (positive for T cells), along with expression of cell surface markers CD4, CD8α and CD8β and intracellular secretion of IFNγ (Figure 10B) and IL-17A (Figure 10C).

**Figure 11:**
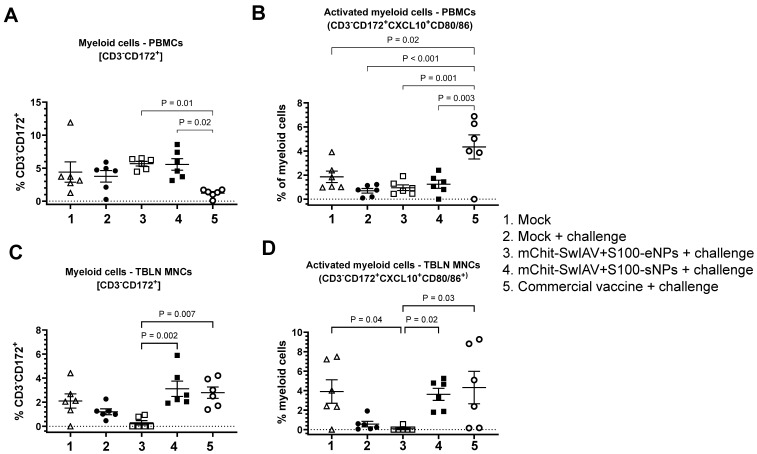
Frequencies of myeloid cells in PBMCs and TBLN MNCs of mChit-SwAIV + S100-NP vaccinated pigs challenged with a heterologous virus. (**A**,**B**) PBMCs and (**C**,**D**) TBLN MNCs of pigs stimulated in vitro with CA09-H1N1 virus. Cell frequency was determined by flow cytometry. Error bars indicate means ± SEMs of 5–6 pigs. The *p* values between groups (*p* < 0.05) were determined by two-way ANOVA with Tukey’s multiple comparisons post-hoc test.

The frequencies of total myeloid cells (CD3^−^CD172^+^) in PBMCs were significantly higher in both the types of mChit-NP vaccine groups compared to the commercial vaccine group (Figure 11A). While in TBLN MNCs CD3^−^CD172^+^ cells, frequency was elevated only in mChit-SwIAV + S100-sNP and commercial vaccine groups (Figure 11C). Further, myeloid cell populations were gated for CD3^−^CD172^+^CXCL10^+^CD80/86^+^ phenotype, given their importance in regulation of inflammation and T-cell activation. In PBMCs, the commercial vaccine group had significantly upregulated frequencies of CD3^−^CD172^+^CXCL10^+^CD80/86^+^ cells compared to both the types of mChit-NP vaccinated groups (Figure 11B). In TBLN, both mChit-SwIAV + S100-sNPs and commercial vaccines induced greater numbers of CD3^−^CD172^+^CXCL10^+^CD80/86^+^ cells compared to the mChit-SwIAV + S100-eNP group (Figure 11D). The higher frequency of activated myeloid cells (CD3^−^CD172^+^CXCL10^+^CD80/86^+^) in some vaccinated groups suggests that this specific myeloid cell subtype could be a mature antigen-presenting cells pool in pigs.

### 3.8. Cellular Immune Responses Induced by mChit-NPs Intranasal Vaccine in Pigs

**Figure 12:**
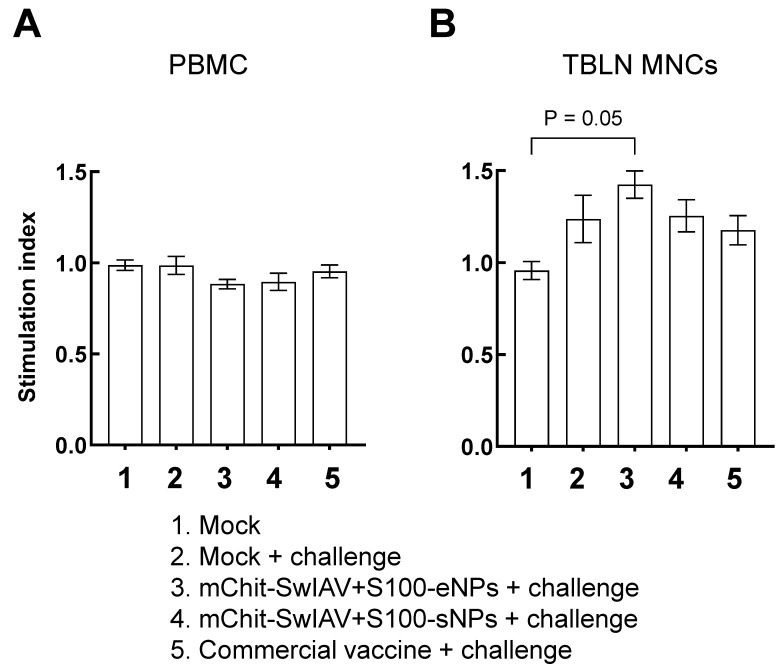
Lymphocyte stimulation index in mChit-SwAIV + S100-NP vaccinated pigs challenged with a heterologous virus. (**A**) PBMCs and (**B**) TBLN MNCs isolated at DPI-6 were stimulated with 0.1 MOI of CA09-H1N1 virus in the presence of recombinant porcine IL-2 for 48 h and analyzed for cell proliferation index. Error bars indicate means ± SEMs of 5–6 pigs. The statistical significance *p* < 0.05 was obtained by analysis of variance (ANOVA) with Tukey’s pair-wise comparison.

To assess the virus-specific lymphocyte proliferation index, TBLN MNCs and PBMCs isolated at DPI-6 were stimulated with the challenge CA09-H1N1 virus. In PBMCs, all the experimental groups had comparable levels of lymphocyte proliferation index (Figure 12A), while the TBLN MNCs of mChit-SwIAV + S100-eNP vaccinates had a significantly higher levels of lymphocyte proliferation index compared to mock control group (Figure 12B).

**Figure 13:**
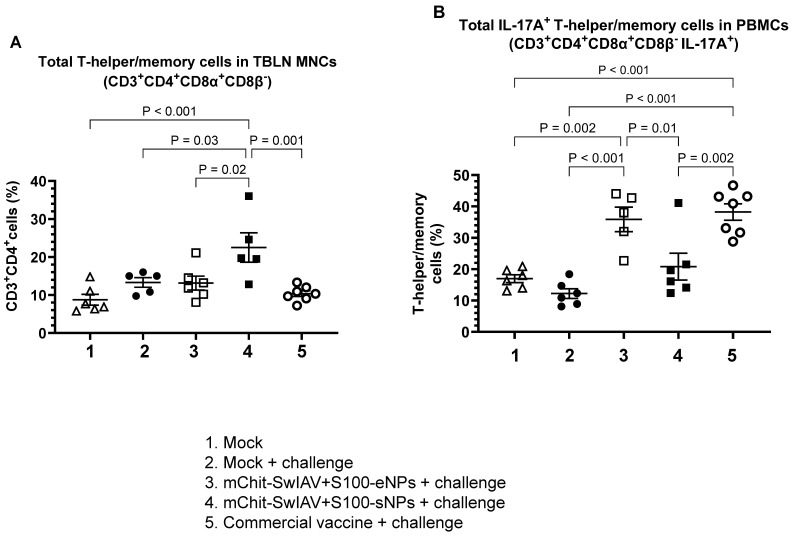
Analysis of T-helper/memory cell frequency in mChit-SwAIV + S100-NP vaccinated pigs challenged with a heterologous virus. (**A**) TBLN MNCs and (**B**) PBMCs isolated at DPI-6 were stimulated with CA09-H1N1 virus and analyzed for the frequency of T-helper/memory cells by flow cytometry. Error bars indicate means ± SEMs of 5–6 pigs. The *p* values between groups (*p* < 0.05) were determined by two-way ANOVA with Tukey’s multiple comparisons post-hoc test.

We analyzed the T-helper/memory cells (CD3^+^CD4^+^CD8α^+^CD8β^−^) frequency and in the TBLN MNCs of the mChit-SwIAV + S100-sNP vaccinated group, we observed significantly higher levels compared to the mChit-SwIAV + S100-eNP and commercial vaccine groups (Figure 13A). Interleukin-17A (IL-17A) is a pro-inflammatory cytokine expressed by activated CD4 T-cells, promoting the B cell response for efficient antiviral activity against influenza virus in the lungs [34]. We detected significantly higher levels IL-17A^+^ T-helper/memory cells in the PBMCs of both the mChit-SwIAV + S100-eNPs and commercial vaccine groups compared to mChit-SwIAV + S100-sNP vaccinates (Figure 13B). These data shows that the mChit-SwIAV + S100-eNP vaccine induced IL-17A-producing T-helper/memory cell responses in heterologous virus challenged animals.

**Figure 14:**
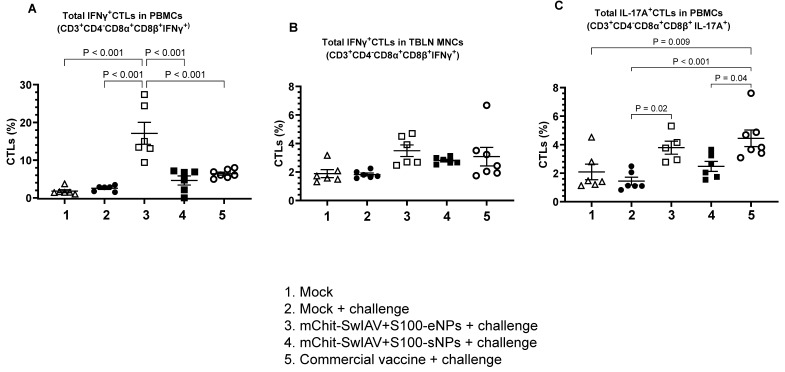
Analysis of IFNγ and IL-17A positive CTLs frequency in mChit-SwAIV + S100-NP vaccinated pigs challenged with a heterologous virus. (**A**,**C**) PBMCs and (**B**) TBLN MNCs isolated at DPI-6 were stimulated with CA09-H1N1 virus and analyzed for the frequency of (**A**) IFNγ^+^ CTLs in PBMC, (**B**) IFNγ^+^ CTLs in TBLN MNCs, and (**C**) IL-17A^+^ CTLs in PBMCs by flow cytometry. The central line in each pig group indicates the mean ± SEM of 5–6 pigs. The *p* values between groups (*p* < 0.05) were determined by two-way ANOVA with Tukey’s multiple comparisons post-hoc test.

Upon identification of the peptide-MHC-I complex, CD8^+^T cells eliminate virus-infected cells and promote IFN-γ production [35]. We analyzed whether immunization with mChit-SwIAV + S100-NPs vaccines elicits CD8 T cell responses specific to the challenge virus. Our data indicate that IFN-γ secreting CTL (CD3^+^CD4^−^CD8α^+^CD8β^+^) frequency was significantly higher in the PBMCs of pigs immunized with mChit-SwIAV + S100-eNPs (~20%) compared to the other two vaccine groups (~6%) (Figure 14A). In TBLN MNCs, the IFN-γ secreting CTLs in mChit-SwIAV + S100-eNPs vaccinates was increased (but not significantly) compared to other vaccine groups (Figure 14B).

We also analyzed activated IL-17A^+^ CTL phenotype ex vivo upon virus restimulation. IL-17A^+^ CTLs recruitment may have a role in protection against influenza A virus infection, regardless of the secretion of IFN-γ [36]. The percentage of intracellular IL-17A^+^ CTLs in both mChit-SwIAV + S100-eNPs and commercial vaccine groups was significantly elevated compared to the mock challenge group, but not in the mChit-SwIAV + S100-sNP vaccinated group (Figure 14C). These data show that intranasal vaccination, especially with mChit-SwIAV + S100-eNP vaccine-induced IFN-γ and IL-17A producing cross-reactive influenza virus-specific CTL and T-helper/memory cell responses.

## 4. Discussion

We evaluated the efficacy of monovalent inactivated SwIAV vaccine adjuvanted with STING agonist ADU-S100 delivered through mChit-NPs in pigs, with vaccine cargo either encapsulated (mChit-SwIAV + S100-eNPs) or surface adsorbed (mChit-SwIAV + S100-sNPs). Delivery of vaccine-active components utilizing mannose conjugated with chitosan on pre-formed NPs require optimization for evaluation of vaccine stability [37]. We characterized the NPs for size distribution, PDI, zeta potential, and LE and LC of the antigen and adjuvant. Since we utilized loading of vaccine proteins on to NPs by two different methods, surface adsorption and encapsulation, each loading was tested separately first on BSA as a model protein. The protocol for surface adsorption of Ags required initial optimization. As per the available literature, this was the first time surface adsorbed vaccine cargo on mChit-NPs was utilized as a mucosal vaccine delivery platform for influenza vaccination.

The surface adsorption approach not only allows the maintenance of the protein structure (similar to that in the target protein), but also enables repetitive antigen presentation to the effector cells [38]. Upon testing of various antigen/nanoparticles (Ag/NPs) ratio, the mass ratio of 1:9 (Ag/NPs) provided a homogeneous size with PDI < 0.5, representing the maximum LE of 74% and 69% for the encapsulation and adsorption loading methods, respectively. This is highly advantageous for vaccine formulation, as a major part of the antigen is bound to the NPs. A high LE percentage relies on the Ag/NPs ratio, with a higher ratio leading to more protein being adsorbed or encapsulated onto the NP [39]. The LE of antigen of interest (inactivated influenza virus) was around 71% and 65% for encapsulated and adsorbed mChit-NPs, respectively. These values were lower than those observed for BSA protein.

This can be rationalized by the isoelectric point (pI) of influenza virus and BSA (pI~6.5 and 4.7, respectively). BSA exhibited strong encapsulation and adsorption to mChit-NPs, likely due to electrostatic interactions between the mannose–chitosan positively charged amino groups with the BSA’s negatively charged carboxyl groups. Conversely, pre-formed mChit-NPs are positively charged at pH 5.7, with a very low LE percentage, explaining its limited binding to the NPs [40], considering that the protein was at a pH below its pI. Therefore, considering the known properties of protein pI values to predict the desired loading efficiency of mChit-NPs is important. Protein encapsulation and/or surface adsorption is a complex process influenced by various other factors [41]. We know that the optimized Ag/NPs weight ratio (1:9) is lower compared to previous optimization studies of maleimide/chitosan NPs (1:6) and regular chitosan NPs’ (1:7) mass ratio, respectively. The maleimide group of maleimide–chitosan NPs has a strong attraction with antigen protein [19], and the positive charge of regular chitosan NPs exhibit greater protein-binding capacity compared to mChit-NPs [39]. Nevertheless, the 1:9 mass ratio of Ag/NPs is higher than mChit-NPs prepared previously by us at a 1:12 mass ratio [14].

In this study, we showed that incorporation of mannose with chitosan NPs at 500 µg/mL had lower cytotoxic effect, with over 75% cell viability. In contrast, the previous study showed that chitosan NPs at 720 µg/mL only caused 30% cell death on treated PBMCs [29]. Consistently, mChit-NPs showed lower cytotoxic effects on HepG-2 and SMMC-7721 cells than chitosan NPs [30]. Therefore, for future studies, it is recommended to utilize mChit-NPs for vaccine delivery which also targets dendritic cells.

The main reason to develop improved mucosal influenza vaccines is to induce robust cross-protective adaptive immune responses at the virus entry site. Antigens in inactivated and subunit IAV vaccines administered via the nasal route are not stable, eventually resulting poor immunogenicity [42]. We combined whole inactivated OH10-H1N2 SwIAV with an agonist ADU-S100 for activating the stimulator of IFN genes (STING) to potentiate the antigen presentation of the vaccine cargo, which was either surface adsorbed or encapsulated on mChit-NPs. The immune responses elicited by vaccine cargo encapsulated versus adsorbed on mChit-NPs delivered intranasal were compared with the responses induced by the conventional commercial SwIAV vaccine.

Intranasal prime-boosting with both types of mChit-SwIAV + S100-NP vaccines suppressed the CA09-H1N1 challenge viral replication in the upper respiratory tract, with mChit-SwIAV + S100-sNP vaccines performing relatively better. This response was presumably associated with cross-reactive influenza-specific memory T cells and mucosal antibody responses induced by the mChit-NPs, leading to reduced viral replication at early time points post-infection (DPI-2 and DPI-4). Although we could not obtain data on virus load in the lungs, the ability to reduce the upper airway viral replication has important implications for both the prevention of influenza infection and its transmission. This result was correlated immunologically with significantly increased frequency of CTLs in the regional TBLN and PBMCs of mChit-SwIAV + S100-eNP vaccinates secreting both IFN-γ and IL-17A, while only IL-17A secreting CTLs in TBLN was increased in the commercial vaccine group. The mChit-SwIAV + S100-sNPs vaccine elicited moderate levels of IFN-γ secreting CTLs.

Activated CD4 T cells play a vital role in initiating B-cell activation and in the generation of antibodies, and they can be categorized into distinct functional groups based on their ability to produce specific cytokines [43]. In swine, a significant proportion of memory CD4^+^ T cells co-express CD8α molecules on their cell surface [44]. Importantly, we showed that T cell immune responses could be induced by the mChit-SwIAV + S100-NP vaccine administered intranasally, and it is cross-protective as indicated by diverse HA gene identification between the vaccine and challenge viruses. Thus, current commercial inactivated SwIAV vaccines provide effective protection against viruses that closely resemble the HA antigenic properties of the vaccine strains [45], while the mChit-SwIAV + S100-NPs vaccine could provide cross-protective immunity in pigs.

This study shows that both the types of mChit-SwIAV + S100-NP vaccine candidates induced potent cross-reactive and strong antibody binding activity and neutralizing activity, with the surface Ag and adjuvant adsorbed vaccine performing relatively better in terms of humoral immune responses and virus clearance. Particularly, the sIgA antibody responses to heterologous and heterosubtypic SwIAV in intranasal mChit-SwIAV + S100-NP vaccine groups demonstrated in this study were promising in terms of the virus clearance from the upper respiratory tract. Consistent with reports showing that nasal IgA protects human influenza infection encompassing low HAI titers [46], the canonical correlates of protection based on HA seroconversion in HAI can be surrogated with neutralizing and sIgA antibody titers specific to HA in the upper airway mucosa [47].

The antibody neutralizing activity in serum was prominently higher than previously demonstrated in humans, swine, and mice vaccinated with an Orf parapox viral vector vaccine [48], split and subunit vaccines [49], and inactivated vaccines [47], respectively. The virus neutralizing activity is correlated with protection after similar influenza A virus challenge infection. Although the mechanism for mucosal immune response after intranasal vaccination has not explicitly been explored yet [47], the improved humoral immune response induced by the mChit-SwIAV + S100-sNP vaccine is potentially based on the viral genomic RNA presence in a whole inactivated virus vaccine with intact Toll-like receptor 7 (TLR7) activation [49], in addition to the potential of other immune sensors such as RIG-I, TLR3, and TLR8 [50] in generating local protective responses in the upper and lower airways and mucosal lymphoid tissues. However, split or subunit antigens possess limited immune stimulation due to the loss of 30–40% of vaccine protein antigens during the preparation process [49,51]. Based on the durability of immune response and protection, the high antibody avidity may be similar to the immune response following natural infection [52]. These observations imply that antibodies targeting the receptor binding site of the HA protein may have a broader cross-reactivity, and the concept of using avidity to expand the range of neutralization activity might apply to various antibodies that target the highly variable surface glycoproteins found in viruses like influenza A virus [53]. Here, we show that intramuscular vaccination produced antibodies of decreased avidity in the respiratory tract, compared to mucosal immunization, which positively correlated with findings in non-human primates when a vaccine was administered directly to mucosal inductive sites. Intranasal vaccination can be more successful in eliciting potent, high-avidity, protective mucosal responses compared to vaccines administered systemically [54]. Finally, strong CTL response in blood after intranasal immunization with encapsulated antigen/adjuvant NPs indicated a strong immunomodulating effect, provided by STING adjuvant in the mucosa-associated lymphoid tissues. This can be based on a concept of compartmentalized immune system responses protecting against infection by mucosal administration of potent vaccines which limit dissemination of the virus systemically [55].

## 5. Conclusions

This study investigated the efficacy of monovalent SwIAV vaccine adjuvanted with the STING agonist ADU-S100 and delivered intranasally to pigs through mChit-NPs employing two vaccine cargo loading methods: encapsulated (mChit-SwIAV + S100-**e**NP) and surface-adsorbed (mChit-SwIAV + S100-**s**NP). Both the NP formulations were extensively characterized for various parameters to derive an ideal particulate vaccine. Notably, for the first time, we pioneered the development of preformed mChit-NPs for delivering surface-adsorbed vaccine cargo for intranasal delivery of an SwIAV vaccine in pigs.

Our findings revealed that the surface adsorption of vaccine cargo on mChit-NPs not only maintained the integrity of vaccine protein, but also enabled repetitive antigen presentation to effector cells, showcasing its potential advantages. We found that the influenza vaccine cargo LE was lower than the loading of model protein BSA, which was attributed to differences in pI. However, our study demonstrated that optimized vaccine Ag/NP weight ratio (1:9) outperformed previous studies, highlighting the importance of considering protein pI values in predicting loading efficiency on mChit-NPs. Further, our research emphasized the lower cytotoxic effect of mChit-NPs, recommending their use for vaccine delivery targeting dendritic cells.

Intranasal administration of both the types of mChit-SwIAV + S100-NPs effectively suppressed viral replication in the upper airways, with the surface adsorbed vaccine performing slightly better. Importantly, the candidate formulations induced cross-reactive, high avidity, virus neutralizing antibodies, with the mChit-SwIAV + S100-sNP vaccine performing relatively better. Furthermore, our findings suggested that high specific IgG antibody avidity induced by mucosal immunization might broaden cross-reactivity, emphasizing the potential superiority of intranasal vaccination in eliciting protective mucosal immune responses. Additionally, our study highlighted the induction of strong CTLs both in blood and regional lymph nodes, indicating the immunomodulating effect of the STING agonist in mucosa-associated lymphoid tissues. Overall, our study contributes valuable insights to the ongoing quest for the development of improved intranasal influenza vaccines, with implications for preventing infection and limiting virus transmission. The need for further research emphasizes understanding long-term immunity, broader strain applicability, and potential translation of this technology into the development of an intranasal influenza vaccine for humans.

## Figures and Tables

**Table 1:** Characterization of mChit-NPs loaded with BSA as an antigen model.

NPs Formulations	Mass Ratio of Ag/NPs	Particle Size (nm)	PDI ^1^	Zeta Potential (mV)	Loading Efficiency (% LE)	Loading Capacity (% LC)
**Blank**	-	246.4 ± 24.0	0.28 ± 0.06	24.4 ± 1.5	-	-
**Encapsulated**	1:9	332.4 ± 20.6	0.32 ± 0.08	19.5 ± 1.4	74.0 ± 5.3	8.3 ± 0.6
**Encapsulated**	1:7	404.7 ± 12.9	0.04 ± 0.04	15.9 ± 1.3	62.5 ± 5.9	8.9 ± 0.8
**Encapsulated**	1:5	569.7 ± 13.7	0.54 ± 0.05	12.9 ± 1.5	54.7 ± 7.4	10.9 ± 0.5
**Adsorption**	1:9	348.7 ± 38.0	0.37 ± 0.09	17.4+1.2	69.4 ± 4.0	7.7 ± 0.5
**Adsorption**	1:7	448.4 ± 52.4	0.44 ± 0.06	14.9 ± 1.0	56.4 ± 3.5	8.0 ± 0.5
**Adsorption**	1:5	783.4 ± 94.1	0.59 ± 0.05	11.2 ± 2.0	50.7 ± 2.1	10.1 ± 0.4

Results are expressed as mean ± SEM (*n* = 3). PDI ^1^ = polydispersity index.

**Table 2:** Characterization of mChit-NPs loaded with OH10-H1N2 antigen.

Formulations	Size (nm)	PDI ^1^	Zeta Potential (mV)
Encapsulated NPs	336.5 ± 33.7	0.36 ± 0.03	18.6 ± 1.8
Surface Adsorbed NPs	396.5 ± 44.1	0.41 ± 0.03	17.4 ± 1.7
Blank NPs	246.4 ± 24.0	0.28 ± 0.06	24.4 ± 1.5

Results are expressed as mean ± SEM (*n* = 3 replicates). PDI ^1^ = polydispersity index.

**Table 3:** Loading efficiency and loading capacity of mChit-SwIAV NPs vaccine.

Formulations	Loading Efficiency (%) *		Loading Capacity (%) **	
	OH10-H1N2 Antigen	Adjuvant ADU-S100	OH10-H1N2 Antigen	Adjuvant ADU-S100
Encapsulated NPs	71.0 ± 6.6	51.0 ± 6.0	3.7 ± 0.3	3.7 ± 0.4
Surface Adsorbed NPs	65.4 ± 6.1	45.8 ± 7.6	3.4 ± 0.3	3.3 ± 0.5
Blank NPs	0	0	0	0

* Loading efficiency—the percentage ratio of the amount of vaccine in the NPs to the total amount of vaccine applied (initial vaccine usage) for NP preparation. ** Loading capacity—the amount of vaccine loaded per unit weight of the NP formulation.

## Data Availability

The datasets used and/or analysed during the current study are available from the corresponding author on reasonable request.

## Supplementary Materials

The following supporting information can be downloaded at: https://www.mdpi.com/article/10.3390/vaccines12060647/s1, Figure S1: The distribution profiles of mChit-NPs loaded with OH10-H1N2 antigen; Table S1: Myeloid cells, IL-17A panel and IFN-γ panel.
