# Supplementary material for: Evaluation of Efficacy of Surface Coated versus Encapsulated Influenza Antigens in Mannose–Chitosan Nanoparticle-Based Intranasal Vaccine in Swine
Source: Vaccines (Basel). 2024 Jun 11;12(6):647. doi: 10.3390/vaccines12060647 (PMC11209417; doi:10.3390/vaccines12060647)
Supplement: Supplementary file 1 [file vaccines-12-00647-s001.zip › vaccines-3027940-supplementary.pdf]

Supplementary data

Formulations	Size	Zeta potential
Encapsulated NPs	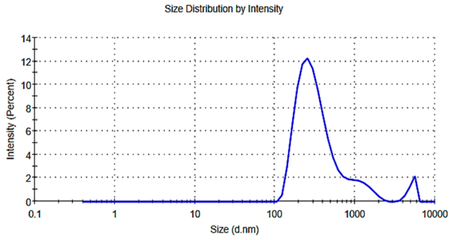	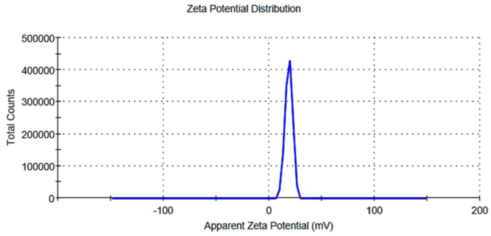
Adsorbed NPs	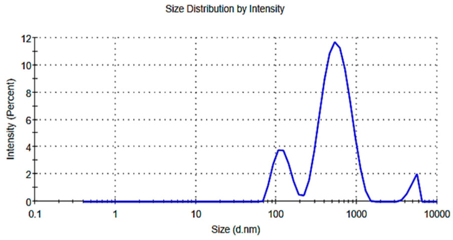	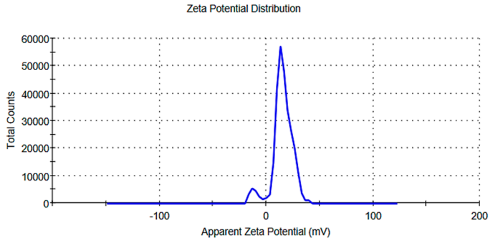
Blank	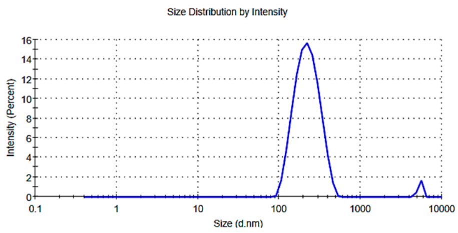	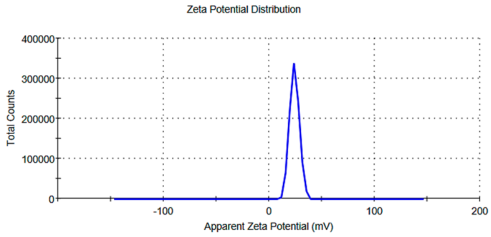

Supplementary Data Figure S1: The distribution profiles of mChit-NPs loaded with OH10-H1N2 antigen.

Supplementary Data Table S1: Myeloid cells, IL-17A panel and IFN-γ panel.

Panel#1-Myeloid cells

Sl.no.	Name	Isotype	Concentration used
1.	Mouse Anti-porcine CD3 AF488 Clone PPT3 (SouthernBiotech)	Mouse IgG1κ	1 µg/ml
2.	Mouse Anti-porcine CD172a-BIOT Clone 74-22-15 (SouthernBiotech)	Mouse IgG1κ	0.5 µg/ml<br>SA PE Texas Red<br>(1:750 dilution)
3.	Anti-porcine CXCL10, Clone 1.4 Labeled with AF647 (Invitrogen)	Mouse IgG1κ	8 µg/ml
4.	Human CTLA-4 Ig PE (Ancel)	Mouse IgG2a	1:100 dilution
5.	Live/Dead<br>AF700 dye (Invitrogen)		1:1000 dilution

Panel#2 IL-17A panel

Sl.no.	Name	Isotype	Concentration used
1.	Mouse Anti-porcine CD3 AF488 Clone PPT3 (SouthernBiotech)	Mouse IgG1κ	1 µg/ml
2.	Mouse Anti-porcine CD8a-BIOT Clone 76-2-11 (SouthernBiotech)	Mouse IgG2aκ<br>SA-PE Texas red<br>(1:750)	0.5 µg/ml<br><br>Streptavidin-PE Texas Red (1:750 dilution)
3.	Mouse Anti-porcine CD8b-UNLB<br>Clone PG164A<br>(mAb -Washington State University)	Mouse IgG2a<br>Goat anti-mouse IgG2a APC/Cy7 secondary antibody (SouthernBiotech)<br>1 ug/ml	0.5 µg/ml
4.	Mouse Anti-porcine CD4 PE-(SoutherBiotech) Clone 74-12-4	Mouse IgG2bκ	0.5 µg/ml
5.	Anti-porcine IL-17A UNLB Clone 1.1<br>Labeled with AF647 (Invitrogen)	Mouse IgG1κ	6 µg/ml
Live/Dead	Fixable AF700 dye (Invitrogen)		1:1000 dilution

Panel#3-IFN-γ panel

Sl.no.	Name	Isotype	Concentration used
1.	Mouse Anti-porcine CD3 AF488 Clone PPT3 (SouthernBiotech)	Mouse IgG1κ	1 μg/ml
2.	Mouse Anti-porcine CD8a-BIOT Clone 76-2-11 (SouthernBiotech)	Mouse IgG2aκ SA-PE Texas red (1:750)	0.5 μg/ml<br><br>Streptavidin-PE Texas Red (1:750 dilution)
3.	Mouse Anti-porcine CD8b-UNLB Clone PG164A (Monoclonal antibody center-Washington State University)	Mouse IgG2a Goat anti-mouse IgG2a APC/Cy7 secondary antibody (SouthernBiotech) 1 ug/ml	0.5 μg/ml
4.	Mouse Anti-porcine CD4 PE (SoutherBiotech) Clone 74-12-4	Mouse IgG2bκ	0.5 μg/ml
5.	Anti-porcine IFN-g AF647 Clone P2G10 (BD Bioscience)	Mouse IgG1κ	1 ug/ml
Live/Dead	Fixable AF700 dye (Invitrogen) (Invitrogen)		1:1000 dilution
